# A challenging sequence of ipsilateral femoral neck fracture, revision total hip arthroplasty, and periprosthetic femoral fracture in a transtibial amputee: a case report

**DOI:** 10.1097/RC9.0000000000001074

**Published:** 2026-10-14

**Authors:** César O. González-Cancino, Daniel A. Castañeda-Rincón, Raúl H. Muñoz-Gómez

**Affiliations:** a Department of Orthopaedic and Traumatology Surgery, Hospital San Lucas, Tuxtla Gutiérrez, Chiapas, México; b Department of Orthopaedic and Traumatology Surgery, Clinext, Tuxtla Gutiérrez, Chiapas, México

**Keywords:** case report, hemiarthroplasty, periprosthetic fracture, total hip arthroplasty, transtibial amputation

## Abstract

**Introduction:**

Patients with transtibial amputation may present gait asymmetry, altered load distribution, and increased risk of falls. Hip fracture treatment and arthroplasty in this population are uncommon and technically demanding.

**Case presentation:**

We report the case of a 59-year-old woman with systemic sclerosis, chronic corticosteroid exposure, osteopenia, and a prior left transtibial amputation secondary to severe vascular disease. Six months after the amputation, she sustained a displaced femoral neck fracture and underwent unipolar hemiarthroplasty at an outside institution. Persistent pain and poor functional recovery prompted conversion to total hip arthroplasty. Intraoperatively, femoral component retroversion was identified. Eighteen months later, she sustained a new fall resulting in a Vancouver type C periprosthetic femoral fracture, which was treated with open reduction and internal fixation. At 24 months after the last procedure, she had satisfactory pain relief, stable implants, fracture union, and functional ambulation with a prosthesis.

**Discussion:**

This case suggests that systemic fragility, altered biomechanics, recurrent falls, and technical factors may have interacted during this patient’s course. The observed sequence should be interpreted as a plausible multifactorial progression rather than a proven causal cascade.

**Conclusion:**

Hip arthroplasty in transtibial amputees requires careful preoperative planning, accurate component positioning, and attention to systemic risk factors such as poor bone quality and fall risk. Nevertheless, given the complexity and unique nature of this case, these recommendations should be considered cautiously, as they are based on a single clinical observation.

## Introduction

In Mexico, lower limb amputation represents a significant public health burden. Vascular disease – including vascular disease related to diabetes mellitus (DM) – is the leading cause, accounting for approximately 81% of all amputations in the country^[^1^]^. Although the present case involves a patient with systemic sclerosis and peripheral vascular disease rather than DM, vascular compromise remains the shared underlying mechanism leading to amputation in both populations.

HIGHLIGHTS

- Transtibial amputation may contribute to altered gait mechanics and an increased fall risk.
- Hip arthroplasty in amputees presents important technical and postoperative challenges.
- We describe an uncommon sequence of ipsilateral femoral neck fracture, revision total hip arthroplasty, and periprosthetic femoral fracture.
- Multiple factors, such as systemic sclerosis, chronic corticosteroid therapy, osteopenia, and recurrent falls, may have contributed to this clinical course.
- Accurate component positioning and individualized surgical planning are essential in high-risk amputees.

Lower-limb amputation is associated with gait asymmetry, functional limitation, and an increased risk of falls. These problems may be amplified in patients with systemic comorbidities that affect bone quality and balance.

Patients with transtibial amputation often rely on compensatory gait strategies to maintain stability, which may increase mechanical demands on the musculoskeletal system. In addition, the absence of distal anatomic landmarks can make hip fracture surgery and arthroplasty technically challenging.

Hip fractures and arthroplasty procedures in amputees are uncommon, and the literature remains limited regarding their short- and long-term outcomes^[^2^]^. We present an unusual clinical sequence in which a transtibial amputee developed a displaced femoral neck fracture, early failure of hemiarthroplasty requiring conversion to total hip arthroplasty, and a subsequent periprosthetic femoral fracture.

This case report was written in accordance with the SCARE guidelines.

## Case presentation

A 59-year-old woman with a 20-year history of progressive systemic sclerosis was treated long-term with colchicine, mycophenolic acid, and deflazacort. She also had osteopenia, with a T-score of *−*1.9, and was receiving antiresorptive therapy with denosumab. Her clinical course was complicated by severe vascular compromise of the left lower extremity and extensive deep venous thrombosis with critical, non-revascularizable ischemia, which ultimately led to a left transtibial amputation (Fig. 1a).

**Figure 1.**
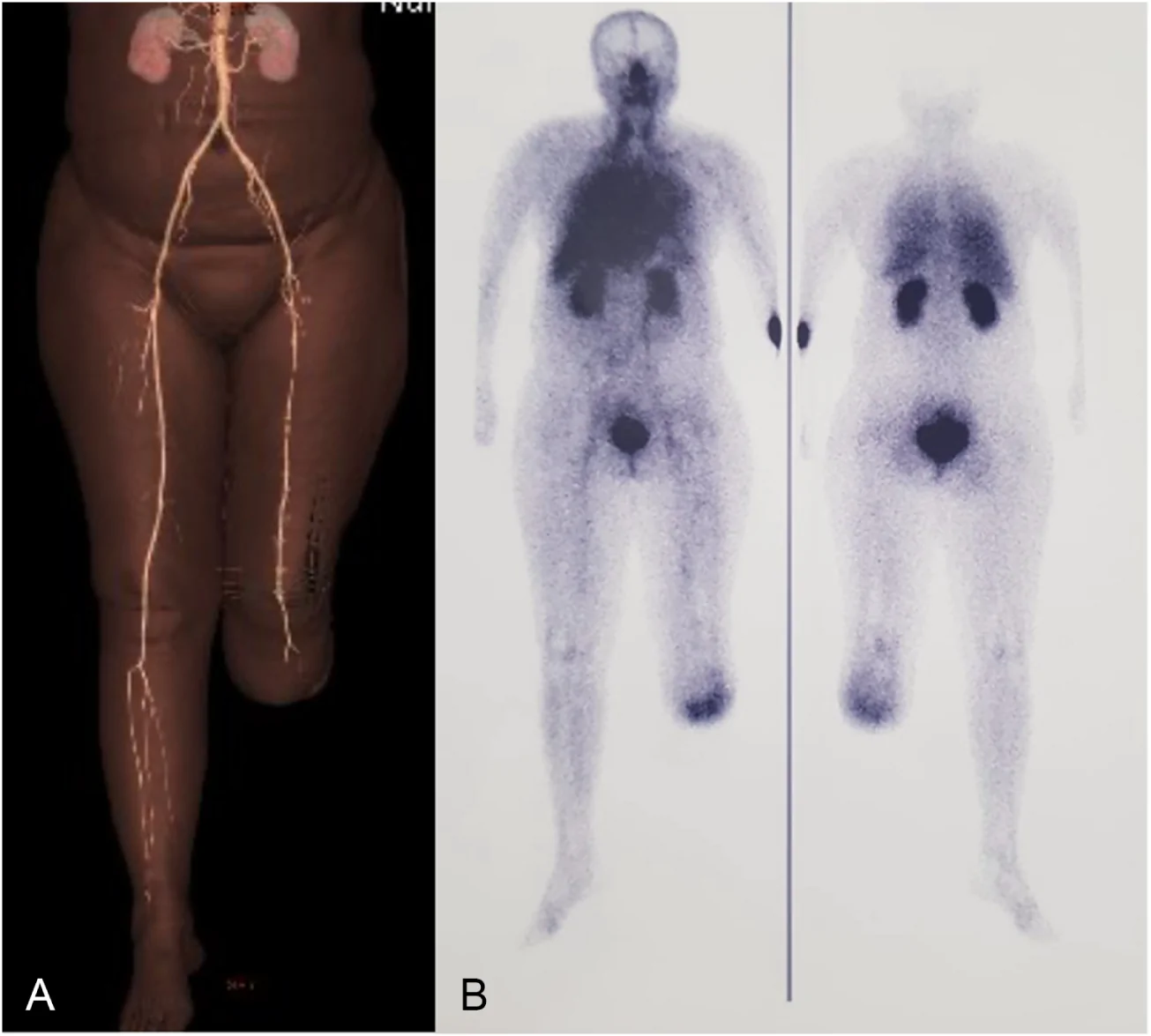
Three-dimensional reconstruction of a computed tomography angiography demonstrating the level of the left transtibial amputation and the vascular status of the residual limb (A). Technetium-99 m (^99^ᵐTc) bone scintigraphy was performed to rule out periprosthetic infection, yielding a negative result (B).

Six months after the amputation, she sustained a displaced transcervical fracture of the left femoral neck after a fall. The patient was using a transtibial prosthesis. Her walking capacity was classified as level K2 (limited ability to walk outdoors) because she was in the early months of physical rehabilitation. She underwent unipolar hemiarthroplasty at an outside institution (Fig. 2a). The indication for conversion was persistent pain [Visual Analogue Scale (VAS): 5 points] and poor functional recovery, with concern for acetabular wear and possible component malposition. Infection and stem loosening were not clinically evident, as confirmed by a negative Technetium-99 m bone scan (Fig. 1b).

**Figure 2.**
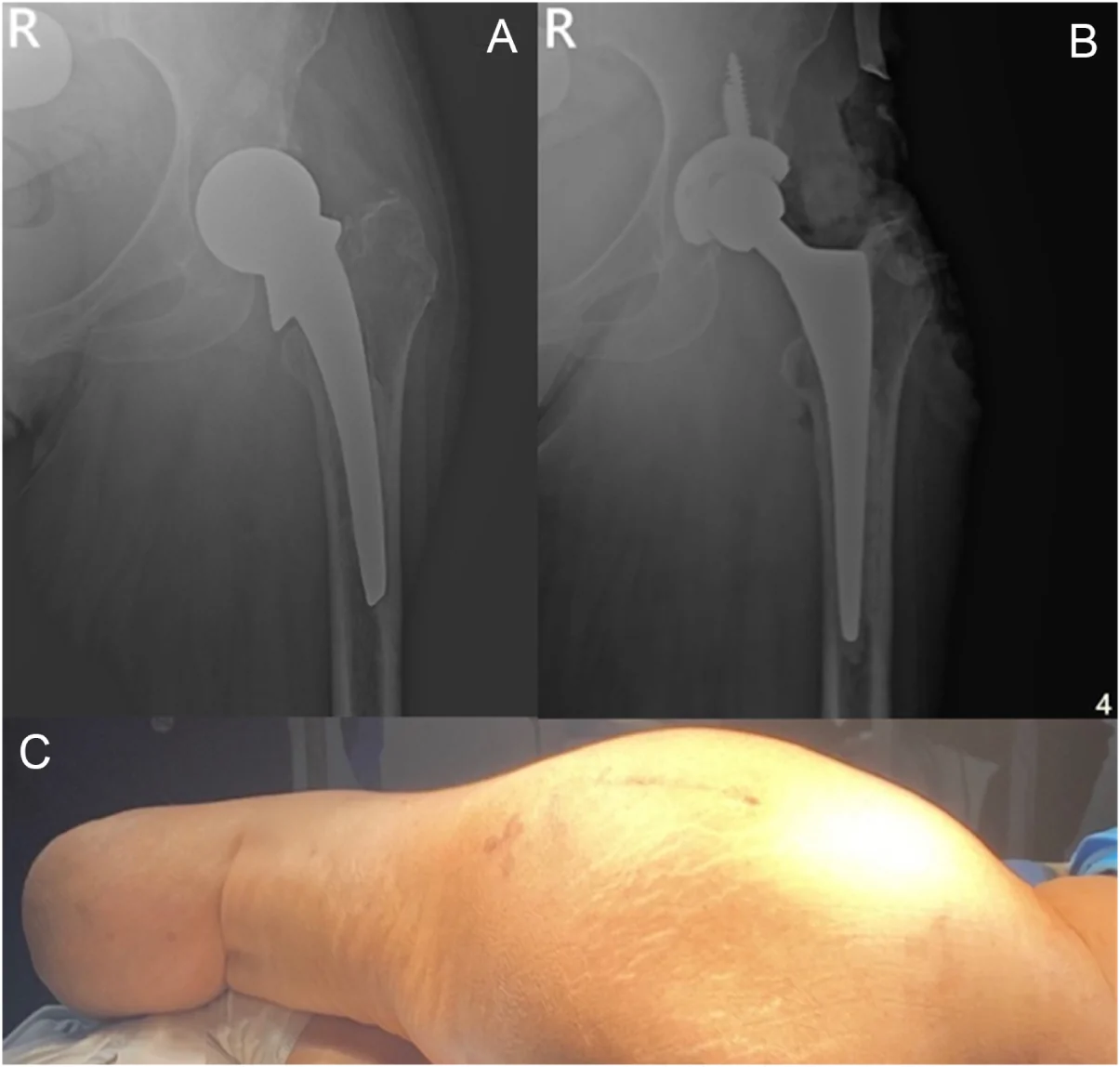
Clinical and radiological sequence of the surgical management. (A) Anteroposterior radiograph showing the initial hemiarthroplasty. (B) Immediate postoperative anteroposterior radiograph following revision to a hybrid total hip arthroplasty. (C) Intraoperative clinical photograph showing the patient in the lateral decubitus position.

Ten months after the initial fracture, she was referred to our institution. Revision surgery was indicated because of persistent pain and functional impairment. The procedure was performed under epidural anesthesia with the patient in the lateral decubitus position through a Hardinge approach (Fig. 2c). Intraoperatively, the femoral component was confirmed to be in approximately 10° of retroversion, corroborating the preoperative concern for malposition. The acetabular surface showed cartilage degeneration. A hybrid total hip arthroplasty was performed with an uncemented acetabular component (48° inclination) and a cemented femoral stem (5° of anteversion) with a 28 mm head. Technically, the cement was not evenly distributed, which facilitated its removal. Manipulation of the femoral stump was challenging; however, the preserved distal femoral condyles – present given the transtibial level of amputation – served as reliable anatomical references for orienting the femoral stem in appropriate anteversion. This construct was selected to optimize stability in a high-risk patient and because of implant availability (Fig. 2b). The use of larger heads or a dual-mobility construct may be considered in similar cases, although these options were not used in this patient.

Postoperatively, rehabilitation included partial weight-bearing for 4 weeks, followed by full weight-bearing, prosthetic fitting, and gait training. Fall-prevention counseling was also provided. The patient resumed ambulation with a prosthesis.

Eighteen months later, she sustained another fall and presented with a Vancouver type C periprosthetic femoral fracture. Open reduction and internal fixation was performed using a distal femoral locking compression plate (Fig. 3a). The plate spanned proximal to the tip of the femoral stem to minimize stress risers. For proximal fixation at the level of the hip prosthesis, the plate was positioned in direct contact with the lateral femoral cortex without placing additional screws or cables; cortical contact was deemed sufficient given the fracture pattern, and this approach was chosen to avoid the risk of iatrogenic fracture in the setting of underlying osteopenia. Fracture reduction was satisfactory, and bone consolidation progressed uneventfully.

**Figure 3.**
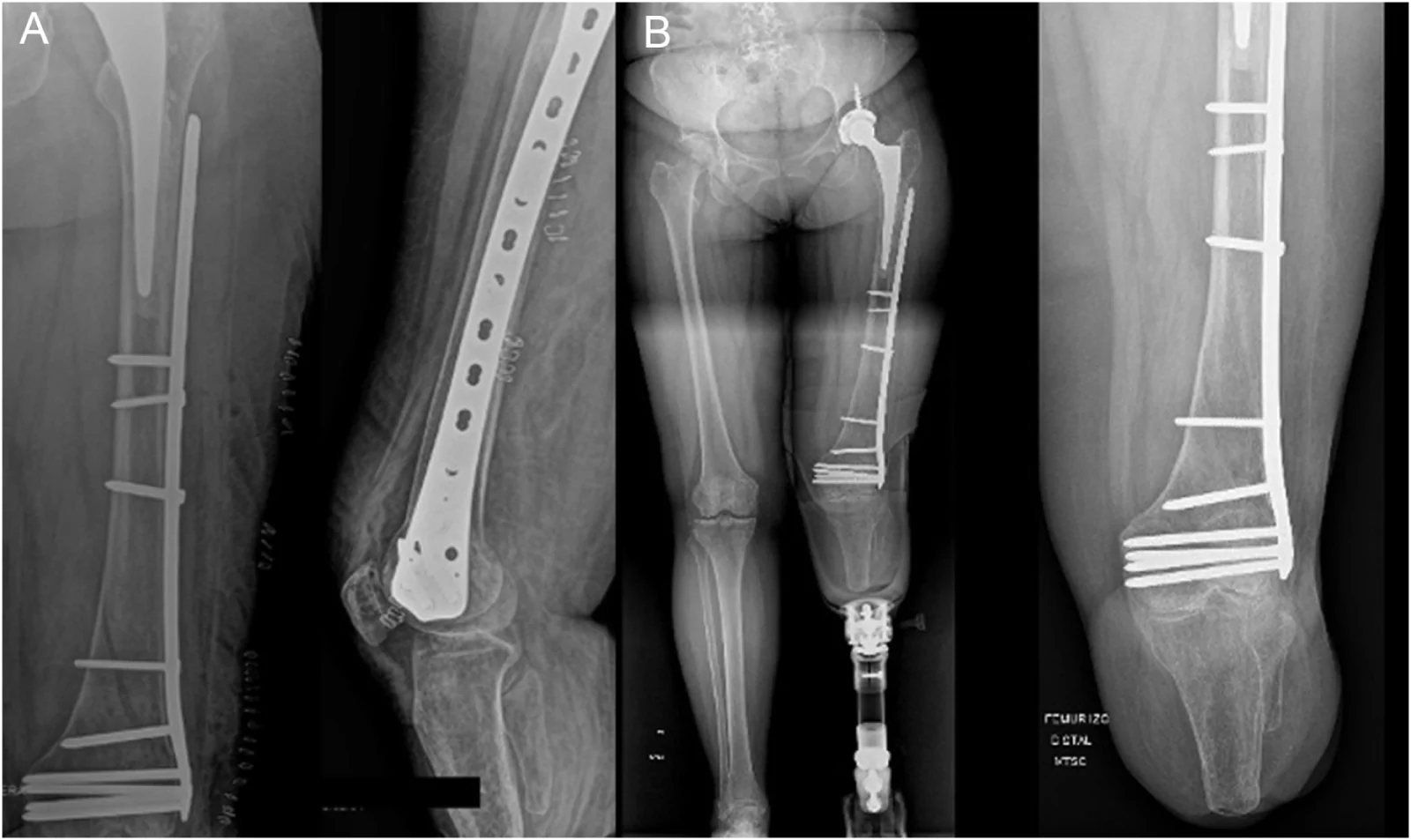
Radiographic evolution of the periprosthetic fracture fixation and long-term follow-up. (A) Postoperative anteroposterior and lateral radiographs demonstrating open reduction and internal fixation (ORIF) of the Vancouver type C periprosthetic fracture using a distal femoral locking compression plate (LCP). (B) Final follow-up radiographs at 24 months (panoramic and localized views) showing complete fracture consolidation and stable prosthetic components without signs of loosening. ORIF, open reduction and internal fixation; LCP, locking compression plate.

At 24 months after the last intervention, the patient was functionally independent and ambulatory with her prosthesis (Level K3: ability to walk outdoors without restriction). Pain had decreased from a preoperative VAS score of 5 to 1 at final follow-up. Radiographs showed complete fracture consolidation and stable implants (Fig. 3b). The Harris Hip Score was 86 points and the Western Ontario and McMaster Universities Osteoarthritis Index score was 18 out of 96, both reflecting satisfactory functional recovery. The patient reported high satisfaction with the final outcome.

## Discussion

This case highlights a complex course in a transtibial amputee with several factors that may have contributed to successive complications. Systemic sclerosis, chronic corticosteroid therapy, osteopenia, severe vascular disease, gait asymmetry, and recurrent falls likely interacted to increase vulnerability to fracture and implant-related problems^[^3^]^.

Individuals with transtibial amputations exhibit altered gait patterns characterized by reduced proprioceptive feedback and a lack of distal ankle control. To compensate for these deficits, patients often adopt a “horizontal impulse” strategy, prolonging movement duration during gait initiation to maintain balance^[^4^]^. This altered biomechanical environment leads to asymmetric anticipatory postural adjustments and compensatory overreliance on the gluteus maximus and medius, which act as the primary stabilizers of the pelvis in the absence of a functional ankle^[^5^]^. Despite continuous advances in prosthetic design, including improvements in materials, socket interface, and biomechanical understanding, restoration of normal gait remains unachievable^[^6^]^. This muscular overload may predispose patients to premature fatigue and compromise the effectiveness of the “hip strategy” for balance control, considerably increasing the risk of falls^[^7^]^.

Although hip fractures in amputee patients are relatively uncommon, their management is technically demanding owing to challenges in positioning, reduction, and implant alignment^[^8,9^]^. The absence of the distal portion of the extremity removes crucial visual and anatomical reference landmarks – such as the alignment of the knee joint and the lower leg axis – which are routinely used by surgeons to gauge native femoral anteversion and component orientation.

The available literature suggests generally favorable outcomes in small series of amputee patients treated for hip fractures, with relatively low complication rates^[^10^]^. However, these studies do not fully address the impact of altered biomechanics on long-term outcomes.

In the present case, initial management with hemiarthroplasty resulted in poor functional recovery, likely influenced by an assumption of low functional demand. In retrospect, this approach may have underestimated the impact of altered gait biomechanics inherent to transtibial amputation, particularly asymmetric joint loading. Additionally, the observed component malalignment may have further contributed to abnormal load distribution. Errors in surgical technique usually manifest as the earliest cause of failure, prompting the need for revision, whereas osteolysis secondary to component wear represents a later-onset event^[^11^]^. Together with the known limitations of hemiarthroplasty in relatively young or active patients, these factors can accelerate acetabular wear and lead to early failure^[^12^]^. These factors likely contributed to the need for early conversion to total hip arthroplasty.

Recent studies confirm that hip arthroplasty in amputee patients can achieve satisfactory functional outcomes, with an average Oxford Hip Score of 40.8, similar to that of the general population. However, the 10-year revision rate in this population (5.9%) has been observed to be higher than the national average for non-amputee patients (4.3%)^[^13^]^. This vulnerability is attributed to atypical ambulatory forces and uneven load distribution, which increase mechanical stress on the implant. In the present case, objective gait analysis was not performed, which limits biomechanical interpretation.

Furthermore, amputee patients have a significantly increased risk of falls. Reported data indicate that up to 50% of individuals with lower limb amputation experience at least one fall annually, with a substantial proportion resulting in injury^[^14^]^. Additional risk factors, including female sex, advanced age, and vascular etiology of amputation, further increase this risk^[^14^]^. Rehabilitation and secondary prevention are also central to treatment. Prosthetic fitting, gait training, and measures to reduce fall risk should be incorporated early.

The later periprosthetic fracture also appears multifactorial. The transition between the implant and native osteopenic bone creates a stress concentration zone^[^15,16^]^, and a new fall likely acted as the immediate precipitating event. This supports the importance of bone health optimization, fall prevention, and careful implant selection in amputee patients^[^17^]^.

Overall, this case demonstrates that outcomes in amputee patients undergoing hip arthroplasty are shaped by the interaction of medical comorbidity, bone quality, gait mechanics, and surgical technique. The sequence observed here should be understood as a plausible multifactorial progression rather than a single causal cascade.

## Conclusion

Hip arthroplasty in transtibial amputees can be technically demanding and may be complicated by poor bone quality, altered gait mechanics, and recurrent falls. In this patient, systemic disease, corticosteroid exposure, osteopenia, and component malposition likely contributed to the clinical course. Careful surgical planning, accurate component positioning, and multidisciplinary prevention strategies are essential in this high-risk population.

## Data Availability

The data supporting the findings of this case report are available from the corresponding author upon reasonable request.

## Research registration unique identifying number (UIN)

Research Registry (researchregistry11957). Registered retrospectively URL: https://www.researchregistry.com/browse-the-registry#home/
